# Clinical Findings among Patients with Respiratory Symptoms Related to Moisture Damage Exposure at the Workplace—The SAMDAW Study

**DOI:** 10.3390/healthcare9091112

**Published:** 2021-08-27

**Authors:** Pia Nynäs, Sarkku Vilpas, Elina Kankare, Jussi Karjalainen, Lauri Lehtimäki, Jura Numminen, Antti Tikkakoski, Leenamaija Kleemola, Jukka Uitti

**Affiliations:** 1 Faculty of Medicine and Health Technology, Tampere University, 33520 Tampere, Finland; lauri.lehtimaki@tuni.fi (L.L.); leenamaija.kleemola@gmail.com (L.K.); jukka.uitti@tuni.fi (J.U.); 2 Department of Phoniatrics, Tampere University Hospital, 33520 Tampere, Finland; sarkku.vilpas@pshp.fi (S.V.); eliina.kankare@pshp.fi (E.K.); 3 Allergy Centre, Tampere University Hospital, 33520 Tampere, Finland; jussi.karjalainen@pshp.fi (J.K.); jura.numminen@pshp.fi (J.N.); 4 Department of Clinical Physiology and Nuclear Medicine, Tampere University Hospital, 33520 Tampere, Finland; antti.tikkakoski@pshp.fi

**Keywords:** moisture damage, mold, dampness, asthma, irritable larynx, respiratory symptoms, laryngeal dysfunction, workplace

## Abstract

Background: Respiratory tract symptoms are associated with workplace moisture damage (MD). The focus of this observational clinical study was patients with workplace MD-associated symptoms, to evaluate the usefulness of different clinical tests in diagnostics in secondary healthcare with a special interest in improving the differential diagnostics between asthma and laryngeal dysfunction. Methods: In patients referred because of workplace MD-associated respiratory tract symptoms, we sought to systematically assess a wide variety of clinical findings. Results: New-onset asthma was diagnosed in 30% of the study patients. Laryngeal dysfunction was found in 28% and organic laryngeal changes in 22% of the patients, and these were common among patients both with and without asthma. Most of the patients (85%) reported a runny or stuffy nose, and 11% of them had chronic rhinosinusitis. Atopy was equally as common as in the general population. Conclusions: As laryngeal changes were rather common, we recommend proper differential diagnostics with lung function testing and investigations of the larynx and its functioning, when necessary, in cases of prolonged workplace MD-associated symptoms. Chronic rhinosinusitis among these patients was not uncommon. Based on this study, allergy testing should not play a major role in the examination of these patients.

## 1. Introduction

Building moisture damage (MD) exposure-associated health effects have been a particular object of research since the 1990s. Epidemiological studies have previously observed that indoor MD exposure is associated with respiratory health effects such as upper respiratory tract symptoms, the development of asthma and asthma deterioration [1,2,3]. So far, these studies have mainly focused on children’s risk of asthma, other respiratory tract symptoms, and MD exposure at home or in schools [4,5,6,7,8], but some previous research has also established a temporal relationship between workplace MD exposure, asthma [9,10,11,12] and rhinitis symptoms [13,14,15].

The above-mentioned epidemiological associations between MD exposure and respiratory tract symptoms and diseases are an important basis of knowledge, but the information on the diagnoses and symptoms of the exposed patients is mostly based on questionnaires. Studies using only questionnaires rather than clinical examinations and tests do not reliably diagnose asthma, and are also unable to diagnose several other diseases related to dyspnea, hoarseness and nasal blockage [16,17].

To our knowledge, only a few previous studies have performed clinical assessments of people exposed to MD at work. Cox-Ganser et al., in their study of the workers of an office building with MD, found abnormal lung function and/or respiratory medication use in 67% of the workers with respiratory tract symptoms [18]. White et al. discovered signs of work-related peak flow changes in serial measurements of workers in an office building with MD [19]. However, no clinical studies have considered the whole respiratory tract system of people with symptoms at workplaces with MD. Not only pulmonary diseases, but also laryngeal dysfunction may cause dyspnea, hoarseness and coughing [20]. Several studies have described laryngeal symptoms that develop at work and are associated with exposure to fumes, odors, or other airborne substances [21,22,23]. Cummings et al. described respiratory tract symptoms, asthma, and rhinosinusitis cases in workers of two office buildings with MD, including two cases of vocal cord dysfunction [24]. Otherwise, no studies on workplace MD exposure being associated with laryngeal dysfunction exist.

The Finnish guideline (2016) for examining a patient with symptoms associated with MD instruct doctors to examine patients according to the symptoms they present by following general diagnostics recommendations. Skin prick tests or allergen-specific IgE measurements are often used with patients with respiratory symptoms because atopy is a known risk factor and a phenotypic feature of many known respiratory diseases. Specific IgE antibody tests for molds are not recommended in primary healthcare, and in secondary healthcare they are usually considered necessary only for patients with severe symptoms that suggest allergy or asthma [25]. The diagnosis of occupational asthma due to MD exposure at workplaces in Finland requires an evaluation of microbial exposure and differential diagnostics in secondary healthcare. Other reasons for referral are a suspicion of asthma with normal PEF monitoring and spirometry in primary healthcare—as additional tests such as methacholine challenge test or exercise test are usually performed in secondary healthcare—or difficult symptoms that affect work ability.

The German–Austrian guideline on medical diagnostics for indoor mold exposure (2016) note that mold exposure may cause mucosal irritation, odor harm and general ill-being. The authors have concluded that indoor molds may cause allergic sensitization, but not as often as other environmental allergens [2]. The existing guidelines are mainly based on studies of the health effects of MD exposure at home, and most of these studies have focused on children. The guidelines may not be ideal for examining adults exposed to MD at the workplace because exposure at work is different to that at home and usually lasts for a shorter time each day. In practice, routine allergy and laboratory tests are often used, but there is no evidence of whether they are effective for workplace MD-exposed patients with respiratory tract symptoms.

In Finland, due to commonly experienced indoor air-associated symptoms and a growing public concern over MDs in buildings and their possible permanent effects on workers’ health, the Prime Minister’s office has set up the project Healthy Premises 2028. Its objectives are to restore public buildings and increase the effectiveness of the treatment and rehabilitation of subjects with indoor air-associated symptoms. The Finnish Institute for Health and Welfare is responsible for executing the project, which aims to enhance the understanding of the effects of indoor environments on health and well-being and to improve the treatment of people with symptoms and illnesses [26].

Improvements in treatment and rehabilitation call for more knowledge on the individual level of the conditions and findings that lie behind MD-associated symptoms, which is why an observational clinical study gathering information systematically was needed.

The focus of this observational clinical study was patients with respiratory tract symptoms associated with MD in the workplace, in order to evaluate the usefulness of different clinical tests in diagnostics in secondary healthcare with a special interest in improving the differential diagnostics between asthma and laryngeal dysfunction.

## 2. Materials and Methods

The study was conducted at Tampere University Hospital, which is a secondary-level referral center for a population of 530,000 and a tertiary-level referral center for a population of 1.1 million people. All patients referred to the departments of Occupational Medicine or Phoniatrics or the Allergy Centre because of symptoms associated with suspicion of MD at their workplace were interviewed as possible study participants between October 2015 and June 2017. We targeted a sample of 100 patients to enable clinical evaluation of patient characteristics. The study inclusion criteria were (1) age between 18 and 65 years, (2) upper and/or lower respiratory tract and/or voice symptoms that are associated with the workplace, and (3) strong suspicion or evidence of MD at the workplace. The criteria on which MD in the workplace was suspected were as follows: (1) indoor air was perceived as moldy or stuffy or otherwise unpleasant, (2) there were signs of MDs (visible mold, moisture spots, discoloration of surface materials, disengaging or blistering of flooring materials, crumbling of wall plastering, water leakages through ceilings (buckets on the floors), and/or loose water on surfaces), (3) renovations because of MDs previously made in the building, and/or (4) information of MD findings had been received from the employer or occupational and health safety personnel. The exclusion criteria were (1) severe illness (e.g., cancer) and (2) pregnancy.

The study protocol has previously been published in detail [27]. In short, the participants were evaluated by specialists in occupational medicine, respiratory medicine and allergology, otorhinolaryngology (ORL) and phoniatrics. The clinical tests of the study patients included blood samples, comprehensive lung function tests (two-week PEF monitoring with measurements twice a day before and after beta-agonist, spirometry with bronchodilation test, methacholine challenge test, a pulmonary diffusing capacity and exhaled nitric oxide (FeNO)), and chest X-ray and cone beam computed tomography (CBCT) imaging of the paranasal sinuses.

Asthma was diagnosed based on symptoms and the demonstration of reversible or variable airway obstruction in lung function measurements: (i) peak expiratory flow (PEF) monitoring, (ii) spirometry with bronchodilation test, or (iii) methacholine challenge test [27]. If the respiratory specialists considered it necessary, the selected patients underwent additional tests such as (high-resolution) computed tomography (CT/HRCT) of the thorax, eucapnic voluntary hyperventilation, or (cardio-pulmonary) exercise tests.

In the ORL specialist’s clinical evaluation, the diagnostic criteria for chronic rhinosinusitis (CRS) were in accordance with the EPOS2012 guideline [28], with symptoms of nasal discharge, nasal blockage, hyposmia, facial pressure/pain or nocturnal coughing for at least 12 weeks and signs of pus in the middle meatus, or pathologic imaging findings in CBCT scans. The CBCTs were also assessed using Lund–Mackay scoring [29].

An experienced phoniatrician assessed the participants’ laryngeal status by indirect video laryngostroboscopy with a 90° rigid telescope (Olympus, Hamburg, Germany), a flexible fiberscope (ENF Type GP, SD video, Olympus, Hamburg, Germany) or a flexible naso-pharyngo videoscope (chip in tip, HD video, Olympus, Hamburg, Germany) with straight and strobe light. Sprayed local anesthesia (Xylocain spray) was used to avoid the gagging reflex. To analyze the video recordings, we used the rpSzene^®^ system (Rehder/Partner GmbH, Hamburg, Germany). We followed international guidelines to diagnose laryngeal dysfunctions and organic laryngeal diseases [30,31,32].

Skin prick testing (SPT) was conducted for common allergen extracts (birch, timothy, mugwort, horse, dog, cat, house dust mite Dermatophagoides pteronyssinus, and latex) (Soluprick SQ, ALK A/S, Copenhagen, Denmark) and *Aspergillus fumigatus* (Soluprick, ALK A/S, Copenhagen, Denmark). These were carried out by trained nurses according to a standardized protocol [33]. The SPT was considered positive, showing sensitization to the allergen, if the wheal size was at least 3 mm larger than the negative control.

IgE antibodies to different fungi that can be found in building structures with MD (*Aspergillus fumigatus*, *Aspergillus versicolor*, *Acremonium kiliense*, *Cladosporium cladosporioides*, *Fusarium moniliformae*, *Penicillium* species, *Stachybotrys atra*, *Trichoderma viridae*) were analyzed using the ImmunoCAP system (Thermo Fisher Scientific, Phadia AB, Uppsala, Sweden) and fluoroenzyme immunoassay (FEIA). Specific IgE ≥ 0.35 kU/L was considered positive.

To control for possible bias related to willingness to participate, based on patient records, the age, sex, line of business, main symptoms, asthma diagnosis, and exposure of the patients who were invited but did not take part in the study were evaluated.

To compare the categorical and continuous variables of the study patients, non-participants and patients with and without asthma, independent sample *t*-tests, Chi-Square, and Mann–Whitney tests were used. Data management and analysis were performed using IBM^®^ SPSS^®^ Statistics Version 25 (2017).

The Ethics Committee of the Pirkanmaa Hospital District approved the study (R14095). All the study participants gave their written informed consent.

## 3. Results

### 3.1. Study Patients

**Figure 1:**
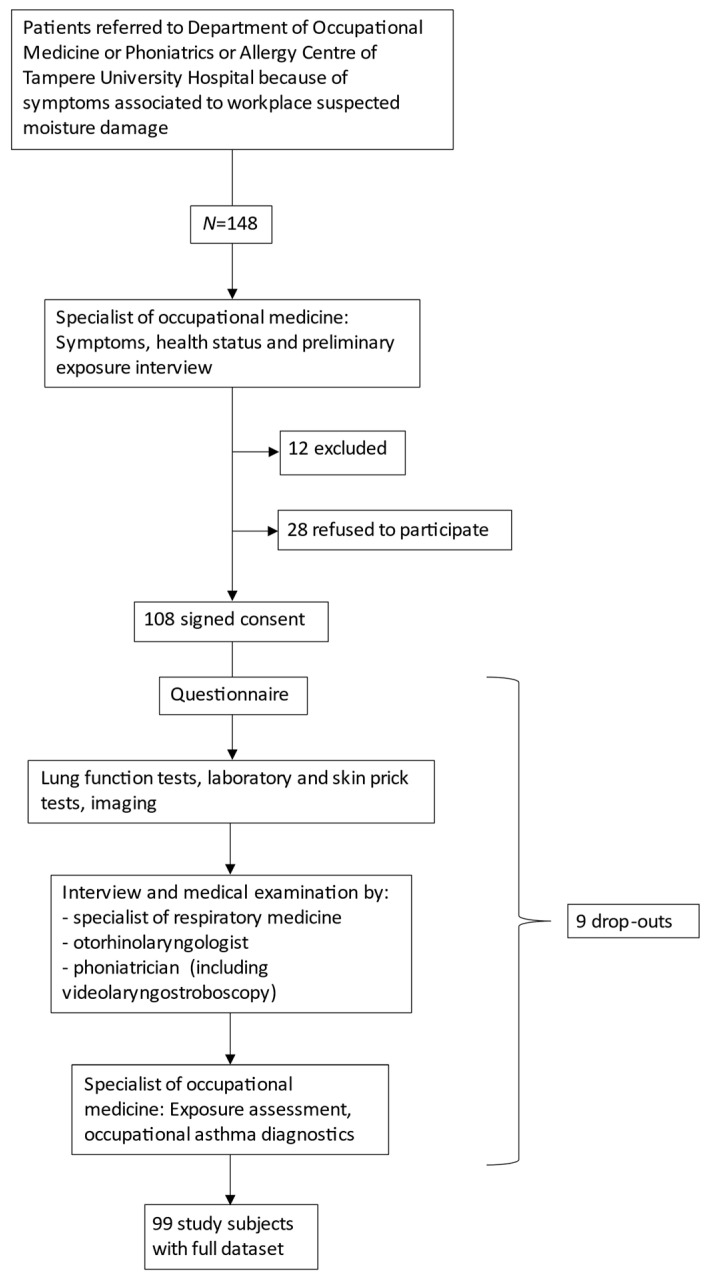
Study design.

To reach a sample size of 100 patients, we interviewed 148 patients between October 2015 and June 2017. The reasons for their referral to secondary healthcare were a suspicion of workplace MD-associated asthma or difficult symptoms associated with workplace MD. In total, 12 patients were excluded as they did not fulfil the inclusion criteria or fulfilled an exclusion criterion, 28 did not want to participate, and 108 gave their consent and participated. Nine patients later withdrew their consent. The final study population consisted of 99 patients (Figure 1), 82 of whom were women and 17 men.

The study patients’ age varied between 20 and 63 years (mean 44 years). Most of the patients (72%) were referred to secondary healthcare by their occupational health physicians. Nine per cent of them were current smokers, one of whom was male. The education personnel (29%) included 23 teachers, and the rest were other school and academy workers. The patients from health services (26%) were nurses, practical nurses, and assistants. The other patients worked in social services (12%), the civil service and national defense (11%), industry or trade (11%), and other lines of business (10%). Based on referral information and anamnesis, 99% of the patients reported hoarseness, 85% a runny or stuffy nose, 92% coughing, and 86% dyspnea at the workplace. Neither cough nor dyspnea were reported by five patients.

### 3.2. Clinical Findings of Respiratory Medicine Specialists

New-onset asthma was diagnosed in 30 patients, and 2 patients had had asthma before employment in the workplace with MD. Of the new asthma diagnoses, fifteen were confirmed by PEF monitoring, nine by findings in both PEF monitoring and spirometry and/or the methacholine challenge test, and six patients were diagnosed as having asthma based on only the spirometry or methacholine test results. The mean FeNO was 21.5 (2.6–63.0) ppb and mean blood eosinophil count (B-Eos) was 206 (20–860) cells/µL in the 30 patients with new-onset asthma, while they were 20.6 (3.3–109.2) ppb and 169 (20–1010) cells/µL, respectively, in the non-asthmatics. Among the new asthma cases, FeNO was >50 ppb in two patients and B-Eos was >300 cells/µL in five patients. Seven new-onset asthma patients had either FeNO or B-Eos. Two patients with a smoking history of at least 15 pack-years had mild airway obstruction, and one of them fulfilled the diagnostic criterion of chronic obstructive pulmonary disease (COPD) (post bronchodilation FEV1/FVC < 0.7).

The patients’ chest X-rays yielded no clinically significant findings. The patients’ pulmonary diffusing capacity of carbon monoxide was normal (mean 98% of predicted, range 73–131%). Ten patients were referred for further diagnostic testing by pulmonary specialists (one for HRCT and bronchoscopy, two for polysomnography, four for exercise testing, one for esophageal pH and impedance recording, and three for additional blood tests). Based on these tests, two patients were diagnosed as having hyperventilation syndrome (one of them also had asthma) and one asthma patient was diagnosed with moderate sleep apnea. A CT of the thorax had been programmed for one patient because of a single 6 mm parenchymal nodule detected 20 months earlier. The nodule was unchanged, and no other pathology was found.

### 3.3. Clinical Findings of Otorhinolaryngology Specialist

Of the 98 patients who underwent the ORL examination, nasal endoscopy revealed that 36 (37%) patients had abnormal findings: 16 (16%) had nasal septal deviation, 9 (9%) had irritated and/or crusty nasal mucosae, 6 (6%) had watery clear discharge, 1 had a nasal septal perforation, and 1 had signs of an acute viral infection. CBCT of the paranasal sinuses was conducted on all the study patients. The EPOS2012 criteria for CRS, including the symptoms and findings from the CBCT images, were met by 11 (11%) patients. Two additional patients had a Lund–Mackay score of over 4 points, but they had no symptoms compatible with CRS. The CBCTs showed that 34 (34%) patients had anatomical abnormalities such as concha bullosa or hypoplastic paranasal sinuses, 28 (28%) had swollen mucosa in their nasal cavities or in paranasal sinuses, 7 (7%) had high dental roots, 5 (5%) had signs of previous endoscopic sinus surgery, and 8 (8%) had fluid retention in their sinuses. There were no significant differences in FeNO and B-eos or atopic sensitization in patients with or without chronic rhinosinusitis (data not shown).

### 3.4. Clinical Findings of Phoniatrician

Organic laryngeal findings were observed in 21 (22%) of the 96 participants who underwent the phoniatrician’s clinical examination. The organic findings were either mucosal (such as laryngitis, vocal fold polyp, node or other mucosal change, vocal fold atrophy) or neurological (paresis or suspected paresis of recurrent or laryngeal superior nerve). Signs of laryngeal dysfunction were observed in 27 (28%) participants: muscle tensions in the larynx in phonation (primary muscle tension patterns), signs of glottic or supraglottic constriction during breathing at rest, or hyperpnea. Vocal cord dysfunction (VCD) was found in three (3%) patients. There were no significant differences in FeNO and B-eos or atopic sensitization in patients with or without laryngeal problems (data not shown).

Table 1 shows the laryngeal and CRS findings in patients with or without asthma. Altogether, 26 (27%) patients who had undergone examinations by all the specialists (*N* = 96) had neither asthma nor any other pulmonary disease, no clinically relevant results in the ORL examination, and no organic or functional laryngeal findings.

### 3.5. Results of Allergy Tests

Atopy, defined as at least one positive SPT reaction in the standard panel (items 1–8 in Table 2), was found in 37%, and sensitization to any pollen (items 1–3 in Table 2) in 34% of the study patients (Table 2).

Comparison of the study patients with asthma (*n* = 32) and without asthma (*n* = 67) revealed no significant differences in the rates of sensitization to any of the tested common allergens—46% vs. 33% (*p* = 0.275)—or *Aspergillus fumigatus*—3% vs. 1.5% (*p* = 1.000). The patients’ allergen-specific IgE showed no sensitization to the different fungi investigated.

### 3.6. Non-Participant Analysis

Of the 28 patients who did not take part in the study, 89% were women. Their mean age was 41, which was three years less than that of the study patients, and varied from 22 to 62 years. The majority (86%) of them reported hoarseness and 31% of them received a new asthma diagnosis when they were examined. The patients who did not take part in the study did not differ statistically significantly from the study patients in terms of age, sex, line of business, symptoms at the workplace, or exposure (data not shown).

## 4. Discussion

In this clinical observational study of patients with respiratory tract symptoms associated with workplace MD, about one third had new-onset asthma. Other pulmonary diseases were uncommon. Functional laryngeal changes were observed in 28% and organic laryngeal changes in 22% of the patients, and they were seen both among asthma patients and non-asthmatics. Most of the patients (85%) had a runny or stuffy nose, but about a tenth had chronic rhinosinusitis, and none had acute bacterial rhinosinusitis. Atopy was equally common among the patients as in the general population, and there were no differences in sensitization to common allergens or MD exposure-associated fungi among the asthma patients compared to among the non-asthmatics.

In our study population, of the individuals with respiratory tract symptoms such as coughing and dyspnea, only about one-third had lower airway dysfunction compatible with asthma. In other words, most of the patients with symptoms that could be interpreted as asthma showed no evidence of variable or reversible airway obstruction. Symptom-based diagnosis of asthma without lung function testing may also cause considerable over-diagnosing of asthma in patients with symptoms related to MD, as many of them probably have laryngeal symptoms only and not true asthma [16,17].

Interestingly, only 7 of the 31 new-onset asthma cases had signs of type 2 inflammation (2 had increased FeNO and 5 had increased levels of blood eosinophils). The proportion of type 2 asthma in our study population was much lower than that usually seen in adult-onset asthma [34,35]. A possible explanation for this is that MD is associated with asthma caused by chronic low-level irritation, which in turn is associated with non-eosinophilic endotypes of asthma [36].

None of the patients showed signs of hypersensitivity pneumonitis (HP; extrinsic allergic alveolitis), which some papers have associated with indoor MD exposure [37,38].

Organic laryngeal changes were more common (22%) here than in a previous study of 78 healthy Finnish female teachers, which found organic laryngeal changes in 14% of the cases [39]. However, no previous research is available on how common functional laryngeal changes are among individuals with or without symptoms. A recent meta-analysis estimated that the prevalence of laryngeal dysfunction among adult asthmatics is 25% [40], which is in line with our study. Respiratory tract symptoms could also be explained by an irritable larynx (IL), a term referring to a hyperreactive larynx leading to increased muscle tension in the laryngeal muscles, dyspnea due to laryngeal constriction, coughing, and voice problems [41]. Our study does not enable us to make conclusions regarding whether MD exposure could cause IL or whether IL is the primary reason for symptoms in a workplace with MD. Either way, in the case of laryngeal disorders, asthma medication does not help or may even worsen symptoms if the larynx is sensitive to irritation [42]. Coexisting with asthma, symptoms of laryngeal origin may be misinterpreted as an insufficient response to asthma treatment. Thorough differential diagnostics and the correct use of asthma medication are thus recommended to avoid unnecessary prolonged symptoms.

Hoarseness was reported by 99% of the patients, but at the first study visit its severity was not evaluated. Hoarseness meant a mild sensation of the voice getting lower for some patients, and complete temporary loss of voice for others. According to previous studies, voice disorders are seen much less in the general population, reported by 17–39% [43,44]. The prevalence of voice disorders among teachers and day care center teachers is high [45,46], but in this study most of the patients were from occupations that are not especially demanding concerning the use of voice. Hoarseness among the patients without laryngeal findings could be associated with other factors than voice strain, such as actual irritative effect of MD exposure or psychological factors [47].

Most of the patients (85%) reported having a runny or obstructed nose in the workplace, but clinical findings in the ORL examination were rather infrequent. None of the patients had acute bacterial rhinosinusitis. Overall, the clinical findings in the upper respiratory tract seemed to be rather modest in the study population, suggesting that most MD exposure-associated nasal symptoms are attributed to irritation by indoor air impurities. Together with nasal congestion, a sensation of paranasal sinus pressure is often reported by MD-exposed patients [24]. Tools are needed to differentiate congestion symptoms from acute or chronic rhinosinusitis among these patients.

In a study of a random sample of 498 individuals aged 26–60 living in Helsinki, Finland, the results were at the same level as those in our study for SPT positivity to birch (19%), horse (5%), and *Dermatophagoides pteronyssinus* (5%), somewhat lower for SPT positivity to timothy (18%) and mugwort (11%), and were higher to cat (20%) and dog (22%). The frequency of sensitization to any pollen (birch, timothy, mugwort) was 33%, which was as common as among our study patients (34%) [48]. Atopy was equally common among the patients with and without asthma. This finding agrees with a review by Mendell et al. (3) which found MD exposure-associated health effects in both allergic and nonallergic persons.

Based on previous research, CRS, allergic rhinitis and asthma are significantly interrelated in the general population [49]. However, within our study population, no such interrelation was found. This may be due to the fact that atopic sensitization or type 2 inflammation was not common among the patients in the current study, and these seemed not to be associated with asthma, CRS or laryngeal findings in this study.

*Aspergillus fumigatus* is an easily sporulating fungus found abundantly in the soil [50]. Sensitization to *Aspergillus fumigatus* is linked to severe asthma [51,52], and the fungus is commonly found in the airways of patients with asthma, regardless of the difficulty of their disease [53]. There is evidence that sensitization to *Aspergillus fumigatus* in individuals with asthma becomes more prevalent with increasing age [54]. In a study by Jaakkola et al. of 21–63-year-olds with recently diagnosed asthma in the same province as our study, the prevalence of serum IgE antibodies against *Aspergillus fumigatus* was 5% in the asthma patients and 2% in the population controls [55]. In another Finnish study, in which the mean age of the participants with asthma was 59, 11% of the asthma patients and 4% of the population controls had positive SPT reactions to *Aspergillus fumigatus* [56]. In our study, sensitization to *Aspergillus fumigatus* among patients was low and in line with previous studies in Finland. In population studies in other European and North American countries, the prevalence of sensitization to *Aspergillus fumigatus* was at the same level (3–6%) [57,58].

Overall, since atopy was equally as common among the patients as in the general population, the SPTs for Aspergillus fumigatus were negative, the specific IgE levels of MD-associated fungi were low, and there were no differences in sensitization to common allergens or MD exposure-associated fungi between the asthma patients and the non-asthmatics, routine testing for possible sensitization among these patients is not useful.

In Finland, located in the subarctic region, it is estimated that 20–26% of hospitals and healthcare centers and 12–18% of schools and kindergartens have significant MD, whereas in office buildings the respective proportion is estimated to be 2.5–5% [6,59]. The higher proportion of teaching and health service personnel among the study patients could at least partly explain the more frequent finding of MD at their workplaces.

About 80% of primary-level teachers in Finland are women [60]. In the Finnish trade union of healthcare employees, 92% of members are women [61]. The high proportion of women in our study is thus at least partly due to more women working in public buildings that have MD. However, there is also evidence that women report more symptoms than men in workplaces with indoor air problems [62], which might also contribute to the female predominance in our sample.

Smoking was somewhat less frequent among the study patients than among the Finnish general population: according to the Finnish Institute for Health and Welfare, in 2017, 13% of 20–64-year-old Finns smoked daily [63]. Therefore, smoking does not explain the symptoms that these patients connect to indoor air problems [62]. In Finnish workplaces, smoking has been forbidden by law since 1995, so exposure to passive smoking at the workplace is not a plausible cause of indoor air symptoms.

Our study sample represented a population with respiratory tract symptoms suspected of being related to workplace MD exposure who had been referred to secondary healthcare. We estimated that a sample of 100 patients would be enough for the clinical evaluation of patient characteristics. The study design and sample size do not enable estimation of whether workplace MD exposure is a risk factor for developing a disease; the main aim of this study was to describe the results and diseases found among individuals who are referred to secondary healthcare due to symptoms associated with workplace MD. Of all the workers exposed to MD at the workplace, only those who have symptoms (either due to MD exposure or co-existing with MD exposure) contact their occupational health services and may eventually be referred to secondary care. Thus, the patients in this study were a selected group of workers, whose probability of having asthma was higher than that of the general population or even of workers exposed to MD. No similar previous studies exist for comparison, and this kind of study design prevents any conclusions from being drawn regarding asthma incidence related to workplace MD exposure. However, based on this study, future research is needed to clarify certain points. Laryngeal findings were common in this patient group, but this finding requires confirmation, e.g., by comparison to laryngeal findings of symptomless subjects. The proportion of type 2 asthma in our study population was much lower than that usually seen in adult-onset asthma. The assessment of whether workplace MD-associated asthma is different from asthma in general calls for follow-up research and studies with sputum samples of bronchial biopsies. Means to recognize patients with true rhinosinusitis amongst the patients with upper respiratory tract symptoms are needed.

This kind of comprehensive clinical study, describing findings in patients with symptoms associated with workplace MD exposure, has not been conducted before. Its strength is in its extensive systematic clinical testing and specialist evaluations of the patients exposed to MD in the workplace, increasing the understanding of symptoms and diagnoses and of whether they relate to the symptoms experienced. The non-participants did not differ significantly from the study patients, which reduces the possibility of bias related to willingness to participate in the study.

## 5. Conclusions

In this study of patients exposed to MD at the workplace and suffering from respiratory tract symptoms, functional or organic changes in the larynx were frequent and common among patients both with and without asthma. Verification of this finding requires further research. Means to recognize patients with true rhinosinusitis and avoid unnecessary treatment with antimicrobial medication due to alleged acute bacterial rhinosinusitis among these patients are needed. However, some suggestions concerning clinical examinations of these patients can be presented already at this point. To avoid unnecessary or symptom-worsening asthma medication, proper differential diagnostics with lung function testing and, when necessary, evaluation of the larynx and its functioning are needed. We conclude that allergy tests do not seem to play a major role in the examination of respiratory symptoms associated with workplace MD exposure.

## Figures and Tables

**Table 1:** Laryngeal and chronic rhinosinusitis findings in patients grouped by asthma diagnosis.

	Asthma (*N* = 32)	No Asthma (*N* = 67)	All (*N* = 99)
Organic or functional laryngeal finding	12 (13% *)	30 (31% *)	42 (44% *)
Chronic rhinosinusitis	4 (13% **)	7 (10% **)	11 (11% **)

* Of the 96 patients who underwent phoniatric examination. ** Of the 98 patients who underwent ORL examination.

**Table 2:** Positive reactions to specific allergens in skin prick tests of study patients.

Allergen	Positive Reactions (%)
1. Birch	20
2. Timothy	23
3. Mugwort	15
4. Horse	5
5. Dog	16
6. Cat	10
7. *Dermatophagoides pteronyssinus*	2
8. Latex	0
9. *Aspergillus fumigatus*	2

## Data Availability

The data presented in this study are available on request from the corresponding author.
